# Linking DNA damage and senescence to gestation period and lifespan in placental mammals

**DOI:** 10.3389/fcell.2024.1480695

**Published:** 2024-09-30

**Authors:** Vijay Pratap Singh, Pushpendra Singh

**Affiliations:** ^1^ ICMR-National Institute of Research in Tribal Health, Jabalpur, Madhya Pradesh, India; ^2^ AcSIR Faculty of Medical Research, New Delhi, India

**Keywords:** placenta, parturition, senescence, lifespan, gestation, genomic instability

## Abstract

The mechanism that synchronizes the timing of parturition remains a mystery. Each mammalian species has a specific duration of gestation that is determined by integrated interactions among the mother, placenta, and fetus. Senescence is primarily driven by DNA damage and is one of the critical factors influencing both parturition and lifespan. In this study, we investigated senescence as a physiological process during pregnancy and observed a gradual physiological increase in senescence in the maternal decidua and placental cells with gestation. This increase in senescence was associated with a gradual physiological increase in DNA damage during gestation. An analysis of the AnAge dataset revealed a positive correlation between the gestation period and maximum lifespan across 740 mammalian species. This finding supports the hypothesis that the rates of DNA damage and senescence may impact both the gestation period and lifespan. We suggest that the relationship between gestation period and lifespan in mammals is mediated by species-specific rates of DNA damage and senescence, necessitating further explorations into their causal roles.

## 1 Introduction

The timing of birth is a major determinant of evolutionary fitness in viviparous species, and there are large variations in the gestational lengths across different mammals. Determination of the gestation period is a complex process involving coordinated interactions among the mother, fetus, and transiently formed organ known as the placenta (Cross, 2005; Burton and Fowden, 2015; Hemberger et al., 2019). Fetuses born preterm (before 37 weeks) have high mortality rates and may develop several neurodevelopmental as well as cardiac abnormalities, whereas fetuses born post-term (after 42 weeks) can pose real threats to the lives of both the mother and fetus (Galal et al., 2012; Singh et al., 2015; Ohuma et al., 2023). Every year, around 13 million babies are born preterm, constituting almost 9.9% of all births globally, which places a significant economic burden on society as most of these preterm babies suffer from long-term disabilities (Ohuma et al., 2023). Despite substantial efforts and extensive research, the molecular mechanisms of parturition (i.e., the action of giving birth to offspring) are not understood fully, even though they are critical determinants of the perinatal outcomes. Fetal organ maturation signals and endocrine signals cannot fully explain the general mechanisms of parturition across all mammals. Changes in the hormonal milieu at term, such as increased estrogen, oxytocin, and cortisol, along with decreased levels of progesterone can cause COX gene expression and prostaglandin synthesis (Challis et al., 2000). Systemic progesterone withdrawal or addition of oxytocin alone is not found to be solely responsible for inducing parturition in various mammalian species and model organisms (Nishimori et al., 1996; Roizen et al., 2008; Hirota et al., 2010). Interestingly, the administration of exogenous prostaglandins in many of the examined species induced abortion and parturition (Casey and MacDonald, 1988; Gibb, 1998; Pierce et al., 2018). These results suggest that activation of COX and its downstream genes are essential for initiating parturition in many species. Recent studies have suggested the role of senescence in activating COX and inducing sterile inflammation in the maternal decidual, placental, and fetal membranes, which in turn could activate prostaglandin synthesis and parturition (Hirota et al., 2010; Menon et al., 2016; Velicky et al., 2018; Singh et al., 2020). These studies also suggest that premature activation of senescence could cause preterm birth.

DNA damage is the main driver of senescence and plays a central role in regulating its processes (Schumacher et al., 2021). Both DNA damage and senescence are critical determinants of species aging, and species with robust DNA damage repair mechanisms tend to have extended lifespans and slower rates of senescence (Maynard et al., 2015; Yousefzadeh et al., 2021). This begs the question of why different mammals have different lifespans and large variations in their gestation periods. Moreover, is there a relationship between lifespan and gestation period? As both lifespan and gestation period are regulated by senescence, we hypothesize that mammals with robust DNA damage repair mechanisms would have both longer lifespans and longer gestation periods. In the present work, we examine and discuss the above fundamental questions from this perspective. We also systematically analyze senescence and its main driver (DNA damage) for different gestational stages in mouse pregnancy. Using the AnAge dataset, we examine the relationships between gestation period and lifespan in mammals. We propose that DNA damage and senescence may be the key drivers regulating gestation period and lifespan in placental mammals.

## 2 Physiological increases in senescence and DNA damage in murine placenta

**FIGURE 1:**
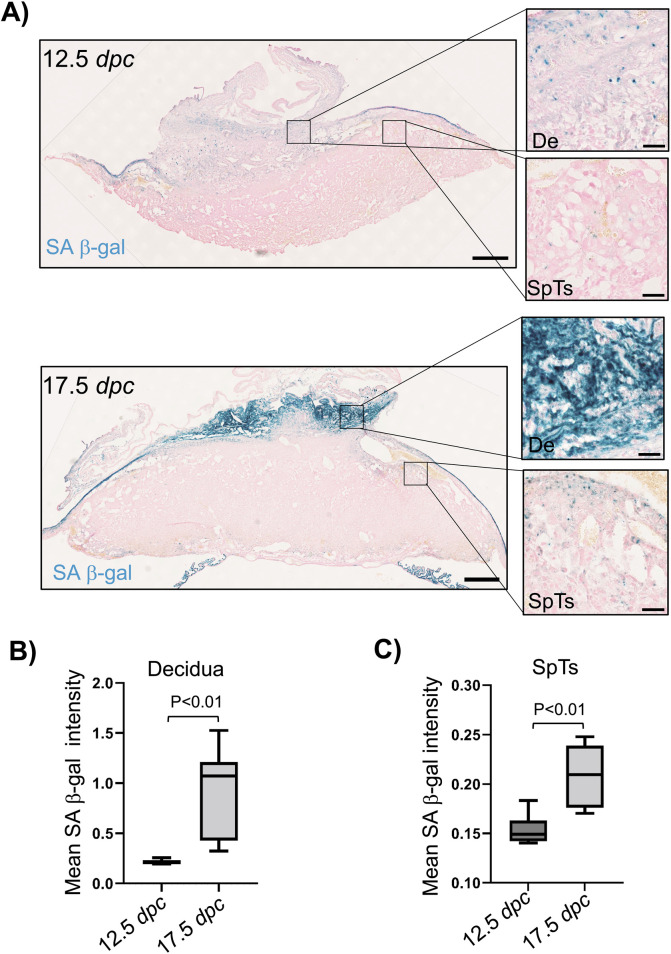
Senescence in the maternal decidua and fetal placenta with gestation in mice. **(A)** The maternal decidua and placenta from C57BL/6J inbred strains were fixed at the 12.5 *dpc* and 17.5 *dpc* stages of pregnancy, and SA β-gal activity was measured as a marker of senescence after X-Gal staining (dark blue). Hematoxylin was used to counterstain all the nuclei. The decidua (De) and spongiotrophoblasts (SpTs) are shown in the magnified images. **(B)** Quantification of the mean SA β-gal intensities in the maternal decidua at the 12.5 *dpc* and 17.5 *dpc* stages of pregnancy (n = 6 placentas). The regions of interest (ROIs) were drawn manually in the decidua, and the mean SA β-gal intensities were quantified. **(C)** Quantification of the mean SA β-gal intensity in the placental SpTs at the 12.5 *dpc* and 17.5 *dpc* stages of pregnancy (n = 6 placentas). The ROIs were drawn manually to mark the SpTs and quantify the mean SA β-gal intensities.

Senescence is typically estimated by measuring the activity of senescence-associated β-galactosidase (SA-β-gal) using X-Gal (a hallmark of senescence) as the substrate (Dimri et al., 1995; Hirota et al., 2010). The number of placental cells is highest at 12.5 days post-coitum (*dpc*) in mouse pregnancy (Eaton et al., 2020), suggesting that there is minimal cell division after 12.5 *dpc* and that the senescence cells do not divide. Thus, we decided to analyze senescence at this time point as well as at a later time point, such as 17.5 *dpc*. We focused on the decidual cells derived from the mother’s endometrium and spongiotrophoblasts (SpTs) of the fetal placenta owing to their distinct morphological features at different stages of pregnancy (Rossant and Cross, 2001). We observed some senescence cells in the placenta and decidua at 12.5 *dpc* (Figure 1A). As the pregnancy progresses, there is a significant increase in the number of senescence cells (Figure 1A). At 17.5 *dpc*, there was an almost 4.4-fold increase in the decidual mean SA-β-gal activity measured through X-Gal staining compared to the decidua at 12.5 *dpc* (Figures 1A, B). In contrast, the increase in mean SA-β-gal activity in the SpTs was only 1.3-fold at 17.5 *dpc* compared to that at 12.5 *dpc* (Figures 1A, C). These results suggest that the major increase in senescence during pregnancy is primarily through the contribution of the maternal decidua and to a lesser extent from the fetal placental cells, which are consistent with previously published results (Bonney et al., 2016).

Because persistent DNA damage is a major driver of senescence, we analyzed the DNA damage at the early stages of pregnancy in mice using γH2A.X immunostaining as the marker of DNA damage (Rodier et al., 2009; Singh et al., 2020). We observed a gradual increase (3.5-fold) in the mean γH2A.X intensity in the maternal decidua from 9.5 *dpc* to 12.5 *dpc* (Supplementary Figures S1A, B). Similarly, the placental SpTs showed a gradual increase (3.8-fold) in the mean γH2A.X intensity from 9.5 *dpc* to 12.5 *dpc* (Supplementary Figures S1A, C). Both the maternal decidua and placental SpTs showed maximum and persistent DNA damage at 12.5 *dpc* since no further increase was observed at 17.5 *dpc* (Supplementary Figures S1B, C). Because the placenta is a polyploid organ and an increase in ploidy may elevate DNA damage during the replication process, we also analyzed the ploidy (nuclear size) in the maternal decidua and placental SpTs at different stages of mouse pregnancy (Singh et al., 2020, 2023). Interestingly, there was no change in the nuclear size in the maternal decidua from 9.5 *dpc* to 17.5 *dpc* (Supplementary Figures S2A, B). However, we observed a 1.6-fold increase in the nuclear size of placental SpTs from 9.5 *dpc* to 12.5 *dpc* (Supplementary Figures S2A, C); furthermore, there was a slight reduction (0.8-fold) in the size of the SpTs from 12.5 *dpc* to 17.5 *dpc*, possibly due to reduction in the overall placental volume at the end of pregnancy, as reported previously (Eaton et al., 2020). Numerous studies have previously shown that increased senescence in the maternal decidua and placental cells can cause preterm birth and fetal growth restriction (Hirota et al., 2010; Perez-Garcia and Turco, 2020; Singh et al., 2020, 2023). Overall, these results indicate that persistent DNA damage during gestation induces senescence in the maternal decidua as well as fetal placental cells and that this senescence is not related to the ploidy of the cells. Based on these results, we propose that these gradual physiological increases in DNA damage and senescence during gestation may be critical drivers of parturition in mammals and that changes in these processes can affect the perinatal outcomes.

## 3 Correlation between the gestation period and lifespan in different inbred mouse strains

**FIGURE 2:**
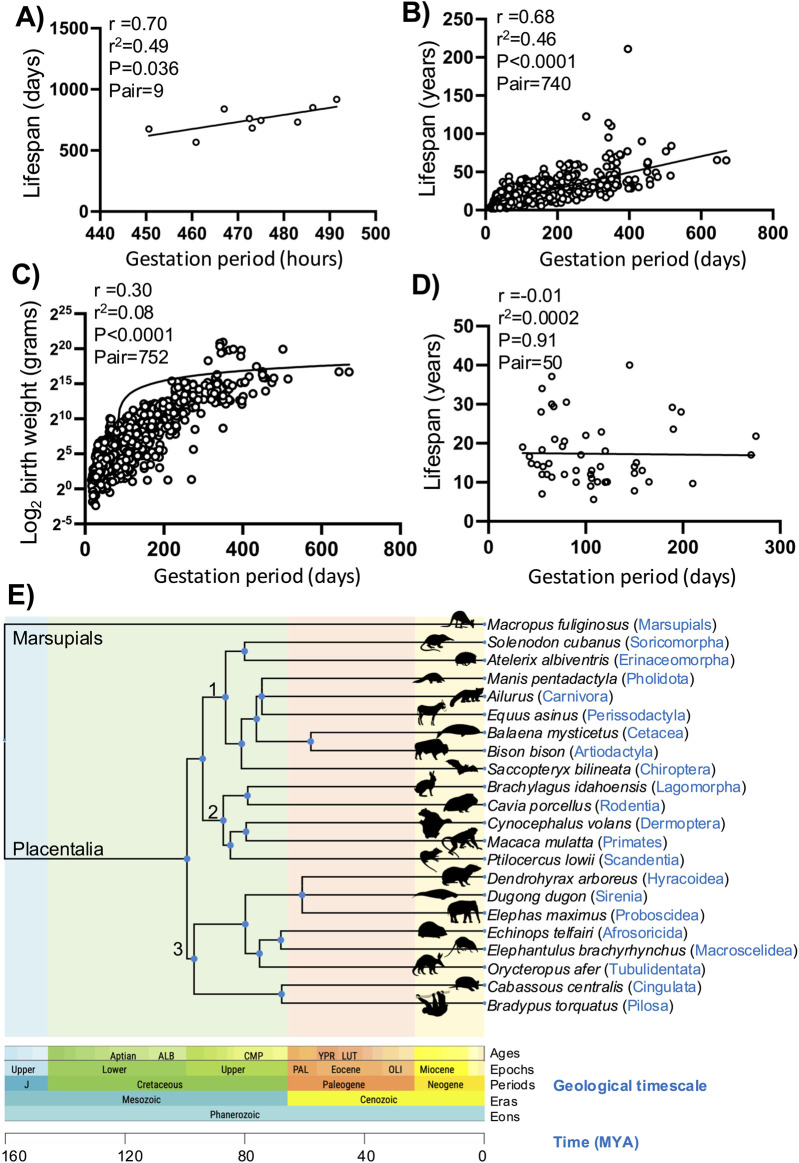
Correlation plots showing the relationships between gestation period, lifespan, and body weight. **(A)** Correlation plot between the gestation periods and lifespans of different inbred strains of mice. The solid line represents the regression curve, and each circle represents a mouse strain. **(B)** Correlation plot between the gestation periods and lifespans for different placental mammals. The solid line represents the regression curve, and each circle represents a single mammalian species. **(C)** Correlation plot between the gestation periods and birth weights for different placental mammals. The solid line shows the regression curve, and each circle represents a single mammalian species. **(D)** Correlation plot between the gestation periods and lifespans in the mammalian order *Chiroptera* (bats). The solid line represents the regression curve, and each circle represents a single bat species. **(E)** Time-tree of the placental mammals. A single representative species was selected from each of the 21 mammalian orders, and MEGA11 (TIMETREE5) was used to plot the evolutionary relationships. A marsupial species was used to illustrate the evolution of the placental mammals; the shaded colors indicate the geological timescale periods, and the distinct clusters are marked as 1, 2, and 3. The images representing each of the mammalian orders were obtained from PhyloPic (http://phylopic.org).

DNA damage and senescence are critical factors in the aging of any organism, and it is now widely accepted that the number of senescence cells increases with age (López-Otín et al., 2013; Schumacher et al., 2021). Somatic mutations accumulate in aged humans and model organisms to promote senescence (Moskalev et al., 2013). Each species has a definite lifespan, and species with longer lifespans have robust DNA repair processes, suggesting lower rates of senescence (MacRae et al., 2015). We propose that the physiological rates of DNA damage and senescence during placental development may reflect the normal aging process in placental mammals. To test this hypothesis, we first analyzed the link between gestation period and lifespan across different inbred strains of mice. Murray et al. (2010) published the gestation periods of 15 inbred strains of mice (AKR/J, CAST/EiJ, KK/H1J, BTBR-T+tf/J, NOD.B10-H2b, DBA/2J, A/J, NZW/LacJ, BALB/cBy, FVB/NJ, C3H/HeJ, 129S1/SvImJ, PWD/PhJ, C57BL/6J, and WSB/EiJ) and showed that strain genetics is a major determinant of the gestational period. We investigated the lifespans of these strains using another dataset published by Yuan et al. (2009). The correlations between gestation period and lifespan for all 15 mouse strains were positive but not significant. However, some of these inbred strains are reported by the Jackson Laboratory to have certain limitations, such as AKR/J that develops lymphoma, KK/H1J that develops diabetes, BTBR-T+tf/J that lacks a corpus callosum, NOD.B10-H2b that shows immune dysfunction, A/J that has a high incidence of lung adenomas, and C57BL/6J that shows seasonal gestational variations, which could affect our analysis; hence, these were excluded from the analysis (Delahunty et al., 2009). By analyzing the remaining nine mouse strains, we observed positive correlations between gestation period and lifespan, with r^2^ = 0.49 and *p* = 0.036 (Figure 2A). These results suggest that mice with longer gestation periods tend to have extended lifespans.

## 4 Correlation between the gestation period and lifespan in mammals

We aimed to analyze the relationships between gestation period and lifespan across all placental mammals. Therefore, we downloaded the aging data from the AnAge resource (Human Ageing Genomic Resources) (De Magalhães et al., 2007; Tacutu et al., 2013). Most of the lifespan records in this dataset are obtained in captivity and tend to be slightly longer compared to species in the wild. The lifespan data for the mammalian order *Chiroptera* (bats) were primarily derived from banding studies, and those for the order *Cetacea* were mostly obtained using indirect methods (De Magalhães et al., 2007). We obtained data on the gestation period, birth weight, and lifespan for more than 740 placental mammals across all 21 mammalian orders. We analyzed the correlations between gestation period and lifespan in these data and observed a strong significant positive correlation, with r^2^ = 0.46 and *p* < 0.0001 (Figure 2B). When we analyzed the correlation between gestation period and birth weight, we found a moderate association, with r^2^ = 0.08 and *p* < 0.0001 (Figure 2C).

We also categorized all placental mammals according to their order. Using MEGA11 (TIMETREE5), we analyzed the evolutionary relationships among all 21 mammalian orders (Figure 2E; Supplementary Figure S3) (Tamura et al., 2021; Kumar et al., 2022; Li et al., 2022) and observed three distinct clusters: 1) *Soricomorpha*, *Erinaceomorpha*, *Pholidota*, *Carnivora*, *Perissodactyla*, *Cetacea*, *Artiodactyla*, and *Chiroptera*; 2) *Lagomorpha*, *Rodentia*, *Dermoptera*, *Primates*, and *Scandentia*; 3) *Hyracoidea*, *Sirenia*, *Proboscidea*, *Afrosoricida*, *Macroscelidea*, *Tubulidentata*, *Cingulata*, and *Pilosa*. We performed correlation analyses between the gestation period and lifespan in all these mammalian orders with more than ten genera in the dataset (Carter and Mess, 2008; Carter, 2012). We found that most orders had positive associations between the gestation period and lifespan, except for *Chiroptera* (bats) (Figure 2D; Supplementary Table S1). Closely related orders such as those in cluster 1 (*Artiodactyla*, *Carnivora*, and *Soricomorpha*) and cluster 2 (*Lagomorpha*, *Rodentia*, and *Primates*) showed strong positive correlations between the gestation period and lifespan (Figure 2E; Supplementary Figure S3; Supplementary Table S1). Interestingly, *Chiroptera* (bats) exhibited that an increase in the gestational period did not increase the lifespan, making them an outlier among all mammalian orders. This finding contrasts with previous observations that bats have higher life expectancies relative to mammals of similar body size (Ricklefs, 2010). Our results suggest that bats should have even longer lifespans given their gestation period. These results indicate that the correlation between gestation period and lifespan is significant. Since both the gestation period and lifespan are regulated by DNA damage and senescence, we propose that the causal roles of these factors in regulating the gestation period and lifespan should be explored.

## 5 Discussion

It is generally believed that traits associated with placental development exhibit antagonistic pleiotropy to support early development but have deleterious effects later in life. Our results suggest that DNA damage and senescence are physiological processes during placental development but have detrimental effects later that contribute to aging. We observed positive correlations between the gestation period and lifespan in placental mammals. Fetal organ maturation and endocrine signals alone could not explain the general mechanism of parturition in all placental mammals. From this perspective, we propose that DNA damage and senescence provide evolutionarily consistent mechanisms to regulate the gestation period and lifespan in mammals. Although our results are correlative and require further validation in several mammalian species, they provide valuable insights into the aging processes of mammals.

Large cohort studies have shown that around 78% of the babies born at or before 27 weeks of gestation develop at least one chronic health condition comparted to 37% for full-term babies (Dance, 2020). There are indications of high mortality rates in preterm babies due to these chronic health conditions, suggesting a decreased lifespan (Dance, 2020). These findings indicate a possible relationship between gestation period and lifespan in humans. Black women in the United States have preterm birth rates that are twice that of Caucasians (Ekwo and Moawad, 1998). Similarly, preterm births are very high in certain populations of Sub-Saharan African countries, such as Nigeria and Kenya, as well as tribal populations in India (Shah et al., 2021; Ohuma et al., 2023). Several factors may contribute to preterm births in these populations, including low income, high infection rates, and potentially genetic causes. There are also indications that these populations have lower life expectancies, but there is a lack of systematic analysis on preterm babies and their health conditions. Understanding genetic factors may thus help improve the quality of life for these populations.

In humans, among the natural conceptions for which the ovulation dates are known, there may be a 37-day variation in the gestation period (Jukic et al., 2013). It is unclear why is there such a large variation in the gestation period in humans. The decidua genotype is always maternal, whereas the placenta and fetal membrane consist of both paternal and maternal genotypes. Our results suggest that the major contributor to senescence is the maternal decidua, but there may also be some contributions from the placenta and fetal membrane. In each pregnancy, the decidual genotype is consistent for a mother but there are large variations in the placental and fetal membrane genotypes because of random segregation of genes during gamete formation. Thus, the gestation length is determined by integrated interactions among the maternal, fetal, and placental genotypes. Given the extensive diversity of human population, such variations in gestational length are expected.

Despite having robust DNA damage repair mechanisms, it is unclear why bats exhibit longer gestation periods but still have shorter lifespans. One possibility is that bats are reservoirs of several viruses, which may contribute to their reduced lifespans (Gorbunova et al., 2020). Given the high load of viruses in bats, they have evolved very strong DNA damage repair pathways (Wang et al., 2011; Huang et al., 2016, 2019); this could be why bats have slower rates of DNA damage and senescence in the maternal decidua and fetal placenta despite their longer gestation periods. It has been shown that the entire PYHIN gene family, including DNA sensors AIM2 and IFI16, are missing in bats along with dampened cytosolic DNA sensors, such as STING-dependent interferon activation (Xie et al., 2018; Gorbunova et al., 2020). This may be another reason for the reduced sterile inflammation and senescence observed in the placentas of bats, despite their longer gestation periods.

Many biologists believe that the rate of aging has a genetic basis; it has also been shown in different strains of mice that the gestation period is genetically determined (Murray et al., 2010). In this report, we propose that DNA damage and senescence could be the most likely links between the gestation period and lifespan in placental mammals. Bats have evolved and robust DNA damage repair processes as well as suppressed DNA sensing mechanisms to dampen inflammation and coexist with viruses. We provide some possible explanations for their unusually long gestation period but require further experimentation to test the DNA damage and senescence in bat placentas at different time points during gestation. Overall, our results suggest that the rates of DNA damage and senescence may be determinants of the gestation period and that perturbations in these processes could lead to preterm births or fetal growth restrictions. We propose that there are critical thresholds for the rates of DNA damage and senescence and that accelerating or decelerating these processes will affect the parturition process. Understanding the molecular regulators of placental DNA damage and senescence will thus aid in elucidating the molecular etiologies of preterm births and parturition.

## Data Availability

The original contributions presented in the study are included in the article/Supplementary Material; further inquiries can be directed to the corresponding author.

## Supplementary material

SUPPLEMENTARY FIGURE S1 DNA damage in the maternal decidua and fetal placenta with gestation in mice. **(A)** The maternal decidua and placenta from C57BL/6J inbred strains were fixed at the 9.5 *dpc*, 12.5 *dpc*, and 17.5 *dpc* stages of pregnancy, and γH2A.X immunostaining was performed to identify the damaged DNA sites using a chromogenic substrate (dark brown). Hematoxylin was used to counterstain all nuclei. The decidua (De) and spongiotrophoblasts (SpTs) are shown in the magnified images. SpTs from the 9.5 *dpc* placenta are highlighted with rectangles, given their small numbers. **(B)** Quantification of the mean γH2A.X intensities in the maternal decidua at the 9.5 *dpc*, 12.5 *dpc*, and 17.5 *dpc* stages of pregnancy (n = 3–6 placentas). The regions of interest (ROIs) were drawn manually in the decidua, and the mean γH2A.X intensities were quantified. **(C)** Quantification of the mean γH2A.X intensities in placental SpTs at the 9.5 *dpc*, 12.5 *dpc*, and 17.5 *dpc* stages of pregnancy (n = 3–6 placentas). The ROIs were drawn manually to mark the SpTs and quantify the mean γH2A.X intensities.

SUPPLEMENTARY FIGURE S2 Nuclear areas in the maternal decidua and fetal placenta with gestation in mice. **(A)** The maternal decidua and placenta from C57BL/6J inbred strains were fixed at the 9.5 *dpc*, 12.5 *dpc*, and 17.5 *dpc* stages of pregnancy, and Feulgen staining was performed to quantify the nuclear areas (light violet). The decidua (De) and spongiotrophoblasts (SpTs) are shown in the magnified images. SpTs from the 9.5 *dpc* placenta are highlighted with rectangles, given their small numbers. **(B)** Quantification of the integrated Feulgen densities in the maternal decidua at the 9.5 *dpc*, 12.5 *dpc*, and 17.5 *dpc* stages of pregnancy (n = 3–6 placentas). The regions of interest (ROIs) were drawn manually in the decidua, and the integrated Feulgen densities (mean intensity multiplied by area) were quantified. **(C)** Quantification of the integrated Feulgen densities in placental SpTs at the 9.5 *dpc*, 12.5 *dpc*, and 17.5 *dpc* stages of pregnancy (n = 3–6 placentas). The ROIs were drawn manually to mark the SpTs and quantify the integrated Feulgen densities.

SUPPLEMENTARY FIGURE S3 Time-tree of placental mammals. At least one representative species was selected from each mammalian family (Supplementary Table S2), and MEGA11 (TIMETREE5) was used to plot the evolutionary relationships. The shaded colors indicate geological time periods, and distinct clusters are marked as 1, 2, and 3. The images representing each of the mammalian orders were sourced from PhyloPic (http://phylopic.org).

SUPPLEMENTARY TABLE S1 Correlation analysis between the gestation period and lifespan for different mammalian orders. Taxonomic orders with a minimum of 10 genera are included in this table.

SUPPLEMENTARY TABLE S2 Dataset downloaded from AnAge. Sheet 1 contains the entire dataset, sheet 2 lists all placental mammalian species, and sheet 3 includes all placental mammals according to their order.
