# Supplementary material for: Linking DNA damage and senescence to gestation period and lifespan in placental mammals
Source: Front Cell Dev Biol. 2024 Sep 30;12:1480695. doi: 10.3389/fcell.2024.1480695 (PMC11471632; doi:10.3389/fcell.2024.1480695)
Supplement: Supplementary file 3 [file Table1.DOCX]

| Gestation length/Lifespan | **Artiodactyla** | **Carnivora** | **Cetacea** | **Chiroptera** | **Lagomorpha** | **Perissodactyla** | **Primates** | **Rodentia** | **Soricomorpha** |
| --- | --- | --- | --- | --- | --- | --- | --- | --- | --- |
| **Pearson r** |  |  |  |  |  |  |  |  |  |
| r | 0.54 | 0.65 | 0.27 | -0.01 | 0.83 | 0.32 | 0.60 | 0.59 | 0.67 |
| 95% confidence interval | 0.41 to 0.64 | 0.55 to 0.74 | -0.06 to 0.56 | -0.29 to 0.26 | 0.50 to 0.95 | -0.22 to 0.71 | 0.47 to 0.70 | 0.49 to 0.68 | 0.22 to 0.88 |
| R squared | 0.29 | 0.43 | 0.07 | 0.0002 | 0.70 | 0.10 | 0.36 | 0.35 | 0.45 |
| **P value** |  |  |  |  |  |  |  |  |  |
| P (two-tailed) | <0.0001 | <0.0001 | 0.112 | 0.913 | 0.0007 | 0.2395 | <0.0001 | <0.0001 | 0.0085 |
| P value summary | **** | **** | ns | ns | *** | ns | **** | **** | ** |
| Significant? (alpha = 0.05) | Yes | Yes | No | No | Yes | No | Yes | Yes | Yes |
| Number of XY Pairs | 139 | 141 | 34 | 50 | 12 | 15 | 119 | 177 | 14 |

**Table S1:** Correlation analysis between gestation period and longevity in different mammalian orders. Taxonomic orders with minimum ten genera are included in this table.
