# Supplementary material for: Linking DNA damage and senescence to gestation period and lifespan in placental mammals
Source: Front Cell Dev Biol. 2024 Sep 30;12:1480695. doi: 10.3389/fcell.2024.1480695 (PMC11471632; doi:10.3389/fcell.2024.1480695)
Supplement: Supplementary file 6 [file Table3.docx]

**Methods:**

**Placenta collection from C57BL/6J mice strain:**

All mice experiments were approved by the Institutional Animal Care and Use Committee of the Stowers Institute for Medical Research (Kansas City, MO) and were performed accordingly.

Mice were housed under controlled temperature and humidity with access of food and water. C57BL/6J females (JAX #000664) were crossed with C57BL/6J male and plugged females were dissected at 9.5 *dpc*, 12.5 *dpc* and 17.5 *dpc*. Placenta were collected in ice cold PBS and processed for downstream applications.

**Senescence Associated β-gal Staining:**

Senescence associated β-galactosidase activity was measured by senescence β-galactosidase staining kit (Cell Signaling #9860) as described previously (Singh et al., 2020). Briefly, placenta at different stages (12.5 *dpc* and 17.5 *dpc*) were collected in PBS at room temperature and fixed in 1 ml, 1X fixative from kit for 3h at room temperature. After fixation placentae were washed three times with PBS and kept in 30% sucrose/PBS buffer overnight at 4°C. The next morning, placenta was embedded in OCT compound (Tissue Tek, #4583) and 16μm cryosections were taken on positively charged slides using Cryostar NX70. Sections were washed once with PBS and once with 1X staining buffer. Next sections were incubated at 37°C for 48h containing 1X staining solution as per kit instructions. After color development, sections were washed with PBS and counterstained with Hematoxylin. Finally, sections were mounted in permanent mounting medium (Vector Laboratories, #H5000) and image were recorded using Olympus VS120 slide scanner with 40X objective.

**Histological analysis and immunohistochemistry (IHC):**

Placenta were dissected at 12.5 *dpc* and 17.5 *dpc* in ice cold PBS. Individual placentae along with mother’s decidua, after removing embryo were fixed in 1 ml 4% paraformaldehyde (Ted Pella, Inc., #18505) overnight at 4°C. The next morning samples were washed three times with 1ml PBS at room temperature and dehydrated in ethanol series to make paraffin blocks. Dehydrated samples were embedded in paraffin blocks and 5μm sections were taken and mounted on positively charged slides (Thermo Fisher Scientific, #12-550-15). Sections mounted on slides were dewaxed and hydrated again with ethanol series and Feulgen staining was performed as described previously (Singh et al., 2020). Briefly, sections were immersed in 5.0 M HCl for 30 minutes and incubated in schiff reagent at room temperature until nuclei were stained. For IHC, dewaxed and hydrated sections were used to perform antigen retrieval using 0.1M citrate buffer pH 6.0 at 95°C for 10 minutes. After antigen retrieval sections were washed with PBS and antibody staining was performed using ImmPRESS Excel Amplified HRP polymer Staining Kit (Vector Laboratories, #MP-7601) with γH2A.X antibody (Cell Signaling # 9718, 1:200 dilution) with overnight incubation at 4°C. Stained sections were counter stained with Shandon™ instant Hematoxylin (Thermo Scientific, #6765015). Sections were mounted with permanent mount medium (Vector Laboratories, #H-5000) and scanned with Olympus VS120 slide scanner using 40X objective. For 9.5 *dpc* stage Feulgen staining and γH2A.X IHC, we used wild type samples (pre stained slides) from our previous study and retook image with 40x objective using Olympus VS120 slide scanner (Singh et al., 2020).

**Quantification and analysis:**

Fiji software ((NIH; <https://imagej.net/Fiji>) was used for image analysis and quantification as mentioned previously (Schneider et al., 2012; Singh et al., 2020). For Senescence associated β-galactosidase activity quantification red absorbance was calculated by taking the negative log of the transmittance intensity. Hand annotated ROIs were used to count how many puncta were present in the absorbance channel and calculate total absorbance for the regions. For γH2A.X quantification, γH2AX positive nuclear areas were found using blue transmittance, and green absorbance used to quantify mean pixel intensities for positive areas. For Feulgen quantifications red absorbance was used to find nuclei using Cellpose and total absorbances and areas per nucleus were calculated from the labeled masks (Stringer et al., 2020).  All plugins and macros are available at <https://github.com/jouyun>. All plots were generated in GraphPad Prism 8. Unpaired student’s *t*-test was used to calculate statistical significance for indicated stage of pregnancy.

**Gestation period and lifespan analysis:**

Gestation period and lifespan data for inbred mouse strains were obtained from Murray et.al., and Yuan et. al., published by the Jackson Laboratory (Yuan et al., 2009; Murray et al., 2010). All 15 inbreed mouse strains were analyzed but inbred strains associated with some adult diseases and mortality along with gestation period variation such as AKR/J, KK/H1J, BTBR-T+tf/J, NOD.B10-H2b, A/J and C57BL/6J were excluded from the analysis. For all mammals, data on gestation period, lifespan and birth weight were downloaded from AnAge resource (Human Ageing Genomic Resources) (Tacutu et al., 2013). MEGA11 (TIMETREE5) was used to analyze evolutionary relationship among all 21 mammalian orders. For each order or family one representative species was selected and TIMETREE5 was used to visualize evolutionary relationships. Images were added using PhyloPic (<http://phylopic.org>) (PhyloPic, n.d.; Tamura et al., 2021; Li et al., 2022). Correlation and linear regression were performed using GraphPad Prism 8 and analysis was performed accordingly.

**References:**

Li, Y., Liu, Z., Liu, C., Shi, Z., Pang, L., Chen, C., et al. (2022). HGT is widespread in insects and contributes to male courtship in lepidopterans. *Cell* 185, 2975-2987.e10. doi: 10.1016/J.CELL.2022.06.014

Murray, S. A., Morgan, J. L., Kane, C., Sharma, Y., Heffner, C. S., Lake, J., et al. (2010). Mouse Gestation Length Is Genetically Determined. *PLoS One* 5. doi: 10.1371/JOURNAL.PONE.0012418

PhyloPic (n.d.). Available at: https://www.phylopic.org/ (Accessed August 21, 2024).

Schneider, C. A., Rasband, W. S., and Eliceiri, K. W. (2012). NIH Image to ImageJ: 25 years of image analysis. *Nature Methods 2012 9:7* 9, 671–675. doi: 10.1038/nmeth.2089

Singh, V. P., McKinney, S., and Gerton, J. L. (2020). Persistent DNA Damage and Senescence in the Placenta Impacts Developmental Outcomes of Embryos. *Dev Cell* 54, 333-347.e7. doi: 10.1016/J.DEVCEL.2020.05.025

Stringer, C., Wang, T., Michaelos, M., and Pachitariu, M. (2020). Cellpose: a generalist algorithm for cellular segmentation. *Nature Methods 2020 18:1* 18, 100–106. doi: 10.1038/s41592-020-01018-x

Tacutu, R., Craig, T., Budovsky, A., Wuttke, D., Lehmann, G., Taranukha, D., et al. (2013). Human Ageing Genomic Resources: integrated databases and tools for the biology and genetics of ageing. *Nucleic Acids Res* 41. doi: 10.1093/NAR/GKS1155

Tamura, K., Stecher, G., and Kumar, S. (2021). MEGA11: Molecular Evolutionary Genetics Analysis Version 11. *Mol Biol Evol* 38, 3022–3027. doi: 10.1093/MOLBEV/MSAB120

Yuan, R., Tsaih, S. W., Petkova, S. B., de Evsikova, C. M., Xing, S., Marion, M. A., et al. (2009). Aging in inbred strains of mice: study design and interim report on median lifespans and circulating IGF1 levels. *Aging Cell* 8, 277–287. doi: 10.1111/J.1474-9726.2009.00478.X
